# Supplementary material for: Vegan and Vegetarian Soups Are Excellent Sources of Cholinesterase Inhibitors
Source: Nutrients. 2024 Jun 26;16(13):2025. doi: 10.3390/nu16132025 (PMC11243061; doi:10.3390/nu16132025)
Supplement: Supplementary file 1 [file nutrients-16-02025-s001.zip › nutrients-3048761-supplementary.pdf]

Supplementary file
Table 1. Soup ingredients, producer codes, names in Polish and English, preservation method, origin

nr	Polish name	English name	Producer Code	Ingredients	Pasteurized/ sterilized	Vegan/ vegetarian
**1**	Barszcz ukraiński	Ukrainian borsch	A	16% potatoes, 12% beetroot, 6% beetroot sour dough, 7% carrot, 5.5% white cabbage, coked white beans, tomato pulp, onion, sugar, cream, carrageenan, vinegar, canola oil, dill, salt, bay leaf, pepper, citric acid.	sterilized	vegetarian
**2**	Dyniowa	Pumpkin soup	B	33%pumpkin, carrot, sweet potato 4.3%, onion, cream, butter, salt, sugar, ginger, tomato concentrate, lime puree, garlic, spices, colander 0.2 %.	pasteurized	vegetarian
**3**	Pomidorowa	Tomato soup	B	Tomato pulp 48%, carrot, tomato concentrate 4 %, onion, celeriac, olive oil, sugar, salt, basil, garlic, oregano.	pasteurized	vegetarian
**4**	Barszcz Ukraiński	Beetroot	C	Beetroot 12.5%, white cabbage 7.5%, beetroot sourdough, potatoes 5%, onion, canned white beans, parsley root, cream, leak, canola oil, salt, tomato concentrate, parsley, starch, vinegar, garlic, sugar, spices.	pasteurized	vegetarian
**5**	Kalafiorowa	Cauliflower soup	C	Cauliflower 20%, potato 17.5%, carrot 4.8%, celery, cream, parsley root, butter, leek, salt, starch, dill, sugar, spices, natural aroma.	pasteurized	vegetarian
**6**	Zupa tajska	Coconut ginger chicken	C	Coconut extract 8%, potato, onion, green pepper 3%, chicken meat 3%, starch, salt, tomato concentrate, peanut paste 2%, canola oil, curry paste, garlic, lemon grass, shallot, galangal, kaffir lime, coriander, cumin, starch, sugar, soy sauce, natural aroma.	pasteurized	chicken
**7**	Zupa jarzynowa	Vegetable with cream	C	Potato 13%, cauliflower 13%, carrot 8%, green beans 4%, green peas 2,2%, cream 2%, carrageen, onion, celery, parsley, salt, canola oil, dill, garlic, spices.	pasteurized	vegetarian
**8**	Ogórkowa	Pickled cucumber	D	Pickled cucumber 21%, potato, carrot, celeriac, onion, parsley root, leak, canola oil, garlic, 0.3% dill, salt, herbs, dried celery.	pasteurized	vegan
**9**	Krem z pomidorów z bazylią	Tomato cream with basil	D	51% tomato pulp, 4.5% tomato concentrate, carrot, potato, onion, celeriac, canola oil, sugar, salt, garlic, 0.3% basil, black pepper.	pasteurized	vegan
**10**	Kartoflanka	Potato soup	E	28% potato, 7% sausage (pork meat, fat, semolina, potato starch, wheat fiber, pea fiber, pork proteins, salt, spices, aromas), carrot, pork meat, leak, celeriac, salt, pork fat, soy protein, spices, herbs, potato starch, dill, garlic, spice extracts.	sterilized	pork
**11**	Wietnamska z kasztanami jadalnymi, limonką i kolendrą	Vietnamese soup with chestnuts, lime and coriander	D	11% coconut extract, onion, potato, carrot, 3% spiced chicken meat, potato starch, salt, aromas, 3% chestnuts, canola oil, 1.5% green curry paste, starch, ginger, garlic, salt, 0,6% lime puree, soy sauce, 0.4% coriander, kaffir lime leaves, spices, aromas.	pasteurized	chicken
**12**	Węgierska gulaszowa	Hungarian goulash soup	F	Vegetables 22% (bell pepper, potato, onion), pork meat 14%, starch, tomato concentrate 1%, wheat flour, salt, pepper, thyme,	sterilized	pork

Supplementary file
Table 1. Soup ingredients, producer codes, names in Polish and English, preservation method, origin

				parsley, spices, yeast extract, aroma, sodium ascorbate.		
**13**	Grochowa z kiełabasą	Pea soup with sausage	F	Vegetables 10% (potato, carrot, celeriac), dried pea 8%, pork sausage 6% (pork meat, salt, modified starch, spices, sugar), smoked pork fatback, modified starch, wheat flour, salt, pepper, marjoram, onions, yest extract, sodium ascorbate.	sterilized	pork
**14**	Pomidorowa z kurczakiem I ryżem	Tomato soup with chicken and rise	F	Tomato pulp 23%, carrots 5%, rice 4%, chicken meat 3%, cream, modified starch, sugar, salt, dried onions, pepper, parsley leaves, yeast extract, sodium ascorbate.	sterilized	chicken
**15**	Żurek z kiełbasą wieprzową	Sour soup	F	Leaven 20% (water, rye flour, garlic, pork sausage 10% (pork meat, salt, modified starch, spices, sugar), sour-cream 2%, modified starch, wheat flour, salt, smoked pork fatback, roasted onions 0.4%(onions, rapeseed oil, wheat flour, salt) garlic, rapeseed oil, marjoram, pepper, yeast extract, aroma, sodium ascorbate.	sterilized	pork
**16**	Tajska	Thai soup	F	Carrot 13%, bell pepper 3%, onion, corn 2%, leak 2%, bamboo shoots, coconut extract 8%, chicken meat 3%, canola oil, wheat flour, tomato concentrate, lemon grass 1.4%, salt, modified starch, sugar, green curry paste 0.3%, shrimp paste, kaffir lime leaves, spices, powdered lemon juice concentrate, sodium ascorbate.	sterilized	chicken
**17**	Zupa krem z zielonego groszku	Green peas cream soup	G	Green peas 31 %, coconut extract 8%, potato, leak, parsley root, garlic, salt, black pepper, wheat flour.	sterilized	vegan
**18**	Zupa krem z włoskich pomidorów	Cream soup with Italian tomatoes	G	Tomatoes 69%, canola oil, sugar cane, soy sauce, lemon juice, garlic, black pepper, salt.	sterilized	vegan
**19**	Zupa krem z pomarańczow ej dyni I mango	Orange pumpkin and mango cream soup	G	Pumpkin 23%, carrot, potato, mango, 4.8%, coconut extract 2.4%, salt, sugar, dried kaffir leave, garlic, lime juice, spices.	sterilized	vegan
**20**	Zupa krem Dyniowa z cynamonem	Pumpkin cream soup with cinnamon	H	Pumpkin 34%, potato, carrot, onion, coconut extract 1.6 %, oranges, sugar, salt, canola oil, oranges puree 0.4%, citric acid, garlic, spices, cinnamon 0.01%.	pasteurized	vegan
**21**	Zupa krem Pomidorowa z bazylią	Tomato cream soup with basil	H	Tomato 51%, carrot, tomato concentrate 5%, celeriac, potato, onion, parsley, sugar, salt, extra virgin olive oil 0.5%, garlic, basil 0.4%, spices.	pasteurized	vegan
**22**	Zupa tradycyjna ogórkowa	Traditional cucumber soup	H	Pickled cucumbers 20%, potato, carrot, celeriac, parsley, onion, leak, canola oil, garlic, dill 0.3%, salt, spices, herbs.	pasteurized	vegan
**23**	Zupa ogórkowa z ziemniakami	Cucumber soup with potato	I	Pickled cucumbers 24%, potato 18%, cream, carageen, wheat flour, carrot 1.5%, spice extracts, salt, glucose, spices, celeriac, modified starch, dill 0.6%, parsley, dried	sterilized	vegetarian

Supplementary file
Table 1. Soup ingredients, producer codes, names in Polish and English, preservation method, origin

				onion, dried parsnip, dried carrot, dried parsley leave.		
**24**	Zupa krupnik z mięsem drobiowym I ziemniakami	Barley soup with poultry and potatoes	I	Potato 11%, chicken meat 7%, onion, barley 4%, carrot 3%, wheat flour, spice extracts, salt, glucose, spices, celeriac, dried parsley 0.1%, pea fiber, poultry proteins, sugar.	sterilized	chicken
**25**	Zupa grochowa z ziemniakami I boczkiem	Dried pea soup with potatoes and bacon	I	Dried boiled pea 17%, potato 16%, pork belly 7%, carrot 4%, spice extracts, salt, glucose, spices, celeriac, dried vegetables, marjoram.	sterilized	pork
**26**	Zupa red curry	Red curry soup	B	Coconut extract 8%, onion, carrot 6%, Pak choi 4%, potato, celeriac, oyster mushroom 2.5 %, red pepper 2%, green pepper 2%, mung bean noodles 2%, tomato pulp, red curry paste 1.5%, canola oil, starch, coriander 0.9%, lime puree, soy sauce, fish sauce, tomato concentrate, spices, garlic, sugar, lime, kafir leave.	pasteurized	vegan with fish sauce
**27**	Zupa ogórkowa z koperkiem	Cucumber soup with dill	B	Potato, pickled cucumber 17.5%, carrot, celeriac, cream, parsley, onion, canola oil, garlic, salt, dill, 0.2%, spices, aroma.	pasteurized	vegan
**28**	Żurek z białą kiełbasą I boczkiem	Sour soup wich whit sousage and pork belly	B	Potato 21%, sour dough from rye flour, onion, white sausage 3.3%, chicken skins, glucose, potato starch, wheat fiber, glucose, celeriac, carrot, cream, smoked pork belly 2%, canola oil, salt, garlic 0.2%, marjoram 0.2%, horseradish, spices, aroma.	pasteurized	chicken, pork
**29**	Zupa krem brokułowy ze szpinakiem	Cream of broccoli soup with spinach	H	Broccoli 36.8 %, onion, potato, spinach 4.3 %, parley root, leek, butter, canola oil, salt, garlic, white pepper, lime concentrate.	pasteurized	vegetarian
**30**	Krem z groszku z miętą	Cream of pea soup with mint	J	Green pea 42%, onion 8.5%, potato, canola oil, spice extracts, spices, garlic cloves 0.4%, salt, dill natural aroma, dried dill, lemon juice concentrate, dried mint 0.08%.	pasteurized	Vegan
**31**	Krem z buraków z mlekiem kokosowym	Beetroot with coconut milk cream soup	J	Cooked beetroot 40%, potato, onion 5%, coconut milk 5%, olive of oil, garlic cloves 0.4%, lemon juice concentrate, spice extracts, spices.	pasteurized	vegan
**32**	Krem z Włoskich przetartych pomidorów	Italian purred tomatoes cream soup	J	Pureed tomatoes 50%, citric acid, onion 8%, carrot, celeriac, potato, tomato concentrate 2%, linden honey, salt, garlic, chili 0.06%, white pepper.	pasteurized	vegan
**33**	Porowa 1 C	Leek soup	labora tory	36% leek, garlic, parsley leaf, parsley root, celeriac, carrot, vegetable broth, white potato, olive oil.	pasteurized	vegan
**34**	Szparagowa 3A	Asparagus soup	labora tory	31,8% green asparagus, 8% onion, 37% white potatoes, 19% cooked parsley, calendula petals, 0.6% parsley leave, 0.2% garlic, vegetable broth, 1,6% olive oil, salt, black pepper.	pasteurized	vegan
**35**	Szparagowa 3B	Asparagus soup	labora tory	31.8% green asparagus, 8% onion, 37% white potatoes, 19% cooked parsley, 0.6% parsley leave, 0.4% garlic, vegetable broth, 1,6% olive oil, salt, black pepper.	pasteurized	vegan

Supplementary file

Table 1. Soup ingredients, producer codes, names in Polish and English, preservation method, origin

**36**	Rokitnikowa 2A	Sea Buckthorn	labora tory	64,7% Apple (*Malus domestica idared*), 18,5% white potato, 9 % sea buckthorn fruit, 0,6% cinnamon bark, 0.6% olive oil, salt, fresh grained black pepper, vegetable broth.	pasteurized	vegan
**37**	Grzybowa 6B	Boletus soup	labora tory	5% boletus (*Imeria badia*), white potato, onion, blackthorn, garlic, vegetable broth, olive oil.	pasteurized	vegan
